# *Radiologia Brasileira:* the constant struggle for its improvement

**DOI:** 10.1590/0100-3984.2020.53.1e1

**Published:** 2020

**Authors:** Edson Marchiori, Valdair Francisco Muglia

**Affiliations:** 1 Editor-in-Chief of Radiologia Brasileira. Email: edmarchiori@gmail.com.; 2 Scientific Director of the Colégio Brasileiro de Radiologia e Diagnóstico por Imagem. Email: valdair.muglia@gmail.com.

Improving the journal **Radiologia Brasileira (RB)** and getting it indexed in international databases has long been a priority for its editors and for the Board of Directors of the Colégio Brasileiro de Radiologia e Diagnóstico por Imagem (CBR, Brazilian College of Radiology and Diagnostic Imaging). Those have also been the objectives of practically all major national and international medical journals^(1)^.

The Brazilian Coordenação de Aperfeiçoamento de Pessoal de Nível Superior (CAPES, Office for the Advancement of Higher Education) recently promoted changes to its graduate program evaluation system, bringing to the national scenario in Brazil what is already a reality in the international scientific publishing community: the growing role of Scopus/Scimago, a database created by Elsevier, which is becoming increasingly more well structured and comparable to the more traditional, more widely used, and until recently unique Web of Science database (formerly owned by Thomson Reuters and now part of Clarivate Analytics), from which the Journal Citation Reports (JCR) is produced on an annual basis. The JCR is the most commonly used citation reference for calculating the impact factor of a journal. The Scimago database has established itself by covering a larger number of scientific publications, including all of those indexed in the Web of Science database. In comparison with that of the Web of Science, the Scimago database covers approximately twice as many journals from three times as many countries^(2)^.

Scientific journals are primarily classified, in terms of quality, by their impact factor (as published in the JCR) or by its Scopus equivalent, the Scimago CiteScore, which is based on the number of citations per document (cites/doc). Both indicators are based on the use of a similar formula: the measure of the impact of a journal is determined by calculating the number of citations received by the articles it published in a given year or number of years^(2,3)^.

Since the indexing of **RB** in the Scopus/Scimago database (in 2012/2013) and in PubMed (in 2015), its Scimago CiteScore has been progressively increasing, from 0.669 in 2014 to 2.868 in 2018. That places **RB** in a privileged position in the Scimago ranking. If we consider the cites/doc for the last two years, it is currently ranked 59th among 328 radiology journals worldwide and is now the highest-impact journal among the 207 indexed medical journals of Latin America^(4)^.

Authors and researchers in the field of radiology, especially those pursuing a master’s or doctoral degree in a graduate program evaluated by CAPES, benefit greatly from the advances in the qualification of our journal, not only in terms of the evaluation of the graduate programs in which they are enrolled but also in terms of their personal evaluations when they apply for research grants from funding agencies. However, everyone can contribute to advancing this process. The only way for the journal to continue to rise in this ranking and to increase its impact further is to raise our awareness regarding the importance of citing **RB** papers in our scientific articles published in other indexed journals. It should also be noted that only citations from the last two years are of interest^(1-3)^. The growing prestige of the journal is evidenced by the fact the number of its citations in other indexed journals rose from 35 in 2014 to 192 in 2018, an increase of 450%.

The **RB** Editors and the CBR Board of Directors are counting on everyone to help the journal play a solid, consistent role on the international stage and, much more importantly, become increasingly more useful to and enjoyable for its readers.

## References

[1] Marchiori E (2015). Mudanças na Radiologia Brasileira para 2015. Radiol Bras.

[2] Marchiori E (2013). Radiologia Brasileira: boas notícias para os pesquisadores nacionais. Radiol Bras.

[3] Kimura ET (2008). ABE&M and impact factor. Arq Bras Endocrinol Metabol.

[4] Scimago Journal & Country Rank https://www.scimagojr.com/journalrank.php?area=2700&country=Latin%20America&order=cpd&ord=desc.

